# Grey Wolf Optimization Inverse Mix Design of Steel Slag Asphalt Mixtures

**DOI:** 10.3390/ma19163497

**Published:** 2026-08-18

**Authors:** Haorui Song, Zhijun Wang, Yangzezhi Zheng

**Affiliations:** Research Institute of Highway, Ministry of Transport, Beijing 100088, China; hr.song@rioh.cn (H.S.);

**Keywords:** steel slag asphalt mixture, mix design, machine learning, SHAP interpretability analysis, optimization algorithm

## Abstract

Pavement mix design for steel slag relies largely on empirical Marshall tests requiring numerous specimens and lengthy cycles. To address this, an inverse mix design (IMD) framework combining machine-learning forward prediction with grey wolf optimization (GWO) was developed. A dataset of 300 samples with 13 input features and 2 output indicators was compiled. Three algorithms—XGBoost, CatBoost, and random forest (RF)—were compared, and model interpretability was analyzed using SHAP and ALE. CatBoost achieved the best overall performance. SHAP identified steel slag f-CaO content and replacement ratio as the dominant factors governing moisture susceptibility. GWO search errors for all three design scenarios were below 0.24%. Laboratory validation showed a mean deviation of 1.02% between target and measured values, confirming the method’s feasibility. The method also supports sustainable pavement engineering by facilitating higher steel slag utilization, contributing to CO_2_ reduction and natural aggregate conservation.

## 1. Introduction

Steel slag, a byproduct of steelmaking, is generated worldwide at approximately 300 million tons per year, making its resource utilization a long-standing priority in environmental protection and civil engineering [1]. Replacing part of the natural aggregate in asphalt mixtures with steel slag can both conserve natural stone resources and reduce the environmental burden associated with slag stockpiling [2,3]. Pavement construction is an important pathway for utilizing steel slag. Its high hardness, excellent abrasion resistance, and strong adhesion to asphalt make it suitable for highway base, subbase, and asphalt surface layers, where it can reduce construction costs and facilitate large-scale use of this bulk solid waste [4,5]. Interest in the use of steel slag in pavement engineering has grown in recent years [6]. Studies have shown that replacing natural aggregate with steel slag can significantly improve the high-temperature stability, low-temperature cracking resistance, and durability of asphalt mixtures, offering a sustainable material option for high-grade highway construction [7,8].

However, the complex mineral composition of steel slag and the substantial variability in its free calcium oxide (f-CaO) content pose major challenges for mixture design, particularly because of strong parameter coupling and the large number of experimental variables [9]. When exposed to moisture, surface f-CaO hydrates to form Ca(OH)_2_, producing a theoretical volume expansion of approximately 128% that may degrade the mixture skeleton and accelerate moisture damage [10]. At the same time, molecular interactions between steel slag and asphalt components can create strong adhesive bonds [11]. Conventional Marshall mix design relies on empirical trial-and-error procedures that require extensive specimen preparation and repeated testing. These procedures are time-consuming and costly and may not achieve an optimal balance among competing performance criteria [12,13]. More efficient, intelligent design methods are therefore needed to enable performance-oriented mix design for steel slag asphalt mixtures.

Machine learning (ML) has been increasingly applied in pavement engineering, offering new approaches to material design and performance prediction [14]. Unlike conventional empirical models, data-driven methods can automatically extract features from complex, high-dimensional material datasets and establish accurate nonlinear relationships between material composition and performance [15,16]. In asphalt mixture design, ML algorithms can efficiently address multivariable coupling and identify solutions that satisfy mechanical performance requirements, economic constraints, environmental considerations, and engineering specifications [17]. Compared with conventional trial-and-error methods, data-driven approaches are better suited to systems with complex multifactor interactions, such as asphalt mixtures. They can rapidly predict critical pavement performance indicators, including high-temperature stability, low-temperature cracking resistance, and moisture susceptibility, thereby supporting pavement design, construction, and maintenance [18]. ML models have shown strong predictive accuracy and generalization across applications ranging from basic indicators such as Marshall stability and flow [19], to long-term performance measures such as fatigue life and rut depth [20], and to assessments of the volumetric stability of steel slag asphalt mixtures [21]. These studies demonstrate the technical feasibility of algorithm-assisted asphalt mixture design.

Nevertheless, several important challenges remain in applying ML to the design of steel slag asphalt mixtures. The complex mineralogy of steel slag and the wide variation in f-CaO content create highly nonlinear effects on moisture susceptibility. Purely data-driven models may therefore fail to reveal the underlying physicochemical relationship between f-CaO hydration-induced expansion and performance degradation [22,23]. Adhesion at the steel slag–asphalt interface involves multiscale interactions, yet most existing models represent these effects using only macroscopic parameters and do not adequately characterize interfacial properties [24]. Moreover, the ‘black-box’ nature of data-driven models limits the physical interpretability of their predictions [25]. This makes it difficult to quantify the pathways and thresholds through which key factors—such as f-CaO content, steel slag replacement ratio, and asphalt–aggregate ratio—affect performance, thereby limiting the value of these models for engineering decisions [26]. A key challenge is therefore to integrate interpretable artificial intelligence with materials science while maintaining predictive accuracy and extracting physically meaningful patterns from empirical data [27,28,29].

Beyond its technical performance, the use of steel slag in asphalt mixtures offers significant environmental and sustainability advantages. Replacing natural aggregate with steel slag reduces the CO_2_ emissions associated with quarrying, crushing, and transporting virgin stone materials. Studies have shown that steel slag can reduce the overall embodied energy of asphalt mixtures by approximately 10–20% depending on the replacement ratio and transport distance [8]. Furthermore, steel slag utilization supports the circular economy by diverting industrial solid waste from landfills and stockpiles, thereby reducing land occupation and potential soil and water contamination. The use of steel slag also reduces the cost of raw materials for pavement construction, as steel slag is often available at lower cost than high-quality natural aggregate in regions near steel production facilities, while simultaneously conserving dwindling natural aggregate resources.

To address these challenges, this study proposes an inverse mix design (IMD) method for steel slag asphalt mixtures that integrates machine-learning-based forward prediction with comprehensive interpretability analysis. The method first establishes forward performance-prediction models that incorporate steel-slag-specific parameters, including replacement ratio and f-CaO content. It then applies SHapley Additive exPlanations (SHAP) and Accumulated Local Effects (ALE) to clarify the nonlinear relationships between input features and pavement performance and to examine multifactor interactions. Finally, the grey wolf optimizer (GWO) is used to determine optimal mix proportions from specified performance targets. The proposed method reduces the repetitive testing required by conventional mix design and improves design efficiency and accuracy, thereby enabling performance-driven mixture design. The framework is broadly applicable and can be extended to the intelligent design of other asphalt mixtures and construction materials containing solid waste. This study supports a transition from experience-based to data-driven material design in pavement engineering and offers a new route for the large-scale, high-value use of bulk industrial solid waste in pavement infrastructure.

## 2. Experimental Methods

### 2.1. Dataset Construction

Data for this study were obtained from both laboratory experiments and published studies to ensure diversity and representativeness. The laboratory experiments used two binders: PG 58-22 base asphalt and SBS-modified asphalt, both produced by Sinopec Chemical Sales Co., Ltd. in Beijing, China. The coarse aggregates were limestone and steel slag. The steel slag was sourced from converter steelmaking at a steel plant and was crushed, magnetically separated, and subjected to accelerated aging for more than 6 months. Limestone was used as the fine aggregate, and limestone powder was used as the mineral filler. Mixtures were designed according to the standard Marshall [22] procedure at five steel slag replacement levels (0%, 25%, 50%, 75%, and 100% by volume of coarse aggregate; this definition is used throughout). The specimens were then prepared and evaluated for pavement performance, yielding approximately 160 valid data records.

Published data were systematically collected from domestic and international journal articles published during the past decade and covered studies from China, the United States, Europe, and other regions. Steel slag sources included converter slag and electric arc furnace slag, while asphalt types included base asphalt, SBS-modified asphalt, and SBR-modified asphalt. Studies were included if they met the following criteria: (1) specimens were prepared using Marshall or gyratory compaction [22]; (2) f-CaO content was determined by chemical titration or XRD; (3) the gradation was SMA; and (4) complete data were available for at least 9 of the 13 input features. After screening, approximately 220 samples with complete data and standardized test conditions were retained.

After the two data sources were merged, duplicate records were removed and the remaining records were screened for validity, leaving 300 samples. The dataset was diverse and representative: the materials originated from multiple regions in China and abroad; the steel slags included converter slag and electric arc furnace slag; the binders included base asphalt and various modified asphalts; and the compaction methods included Marshall and gyratory compaction. The f-CaO content ranged from 0 to 5.5%. Most laboratory data fell between 0.5% and 3.5%, whereas the maximum value of 5.5% came from the literature. This distribution reflects the variability in steel slag properties encountered in engineering practice and provides a sound basis for assessing model generalization.

Input features were selected according to three criteria: (1) frequent reporting in the literature, ensuring data availability; (2) a clear physical or mechanical relationship with mixture performance; and (3) practical measurability in engineering applications. Based on these criteria, 13 input features were selected, covering asphalt properties, mix design parameters, gradation characteristics, and the properties of steel slag and aggregate. The two output variables were moisture-susceptibility indicators: retained stability (MSR) and tensile strength ratio (TSR). Descriptive statistics for the dataset are presented in Table 1.

Data were preprocessed in three steps. First, missing values in some literature-derived features were imputed using distance-weighted K-nearest neighbors (K = 5). For each sample with missing values, the 5 nearest neighbors based on Euclidean distance were identified from the experimental and literature data. A weighted average of the corresponding feature values was then used for imputation, with greater weights assigned to closer neighbors. Compared with simple mean or median imputation, KNN imputation uses correlations among features and therefore better preserves the data structure [30]. Second, outliers were identified using the interquartile range (IQR) method. Values that clearly violated physical principles were removed, and reasonable physical bounds were established for each feature. Third, all continuous input features were normalized to the [0, 1] range using min-max scaling to eliminate differences in scale:

(1) $$x_{scaled}=(x-x_{min})/(x_{max}-x_{min})$$

Finally, the dataset was randomly divided into a training set of 240 samples and a test set of 60 samples at an 80:20 ratio. Five-fold cross-validation was used to evaluate model generalization.

In this study, MSR and TSR were selected as the two output variables because moisture susceptibility is the most critical challenge associated with steel slag utilization in pavement engineering. The hydration of f-CaO causes volumetric expansion that directly compromises moisture resistance, making MSR and TSR the most physically relevant and sensitive performance indicators for evaluating the effects of steel slag properties.

The dataset of 300 samples with 13 input features yields a feature-to-sample ratio of approximately 1:23. To mitigate potential overfitting risk, this study employs several strategies: (1) tree-based ensemble algorithms (XGBoost, CatBoost, RF) with built-in regularization mechanisms to constrain model complexity; (2) 5-fold cross-validation to evaluate model generalization across different data partitions; and (3) rigorous data preprocessing including KNN imputation, IQR-based outlier removal, and physical plausibility checks to ensure data quality. All machine learning algorithms and data analyses were implemented in Python 3.9 using the following libraries: XGBoost 1.7.6, CatBoost 1.2, scikit-learn 1.3.0 for random forest and preprocessing tasks, pandas 2.0.3 for data manipulation, and NumPy 1.24.3 for numerical computations.

### 2.2. Forward Prediction Model

Three machine learning algorithms widely used to predict the performance of pavement engineering materials were selected for systematic comparison [31].

(1) XGBoost (eXtreme Gradient Boosting): XGBoost is an ensemble-learning algorithm based on gradient boosting. It iteratively generates regression trees, with each tree fitted to the residuals (the negative gradient) from the preceding iteration [32]. The XGBoost objective function is defined as:

(2) $$L=\sum_{i}l(y_{i},\;{\hat{y}}_{i})+\sum_{k}\Omega (f_{k})$$

where $l(y_{i},\;{\hat{y}}_{i})$ represents the loss function (mean squared error in this study), $\Omega (f_{k})$ denotes the regularization term for the k-th tree, defined as $\Omega (f_{k})=\Upsilon T+{\frac{1}{2}\lambda \left\|w\right\|}^{2}$, where T is the number of leaf nodes, w is the leaf node weight vector, and Υ and λ are regularization parameters. XGBoost also incorporates techniques such as column subsampling and weighted quantile sketch to further enhance model generalization capability and computational efficiency.

(2) CatBoost (Categorical Boosting): CatBoost is designed specifically for datasets containing categorical variables. It uses ordered encoding to avoid the target leakage associated with conventional target encoding, employs symmetric (oblivious) trees with the same splitting condition at each level to ensure numerical stability during inference, and reduces bias in gradient estimates through an ordered boosting mechanism. These features make CatBoost particularly suitable for engineering problems involving mixed data types [33].

(3) RF (Random Forest): RF is a bagging-based ensemble-learning method. Its procedure comprises three main steps: N training subsets are generated by bootstrap sampling with replacement; a decision tree is constructed for each subset, with m features (m < M) randomly selected from all M features as candidate split variables at each node; and the outputs of all trees are averaged to obtain the final prediction. RF reduces variance through the dual randomization of samples and features, provides an inherent measure of feature importance, and generally performs robustly without extensive hyperparameter tuning [34].

Model hyperparameters were determined through Bayesian optimization [35]. Main parameter search ranges for XGBoost and CatBoost were: $n_{estimators}\in \left[50,500\right]$, ${learning}_{rate}\in \left[0.01,0.5\right]$, and ${max}_{depth}\in \left[3,15\right]$; RF parameters were: $n_{estimators}=300$, ${max}_{depth}=12$, and ${min}_{{samples}_{split}}=5$.

### 2.3. Model Performance Evaluation Metrics

This study employed four metrics to evaluate model performance:

(3) $$R^{2}=1-\sum {\left(y_{i}-{\hat{y}}_{i}\right)}^{2}/\sum {\left(y_{i}-ȳ\right)}^{2}$$

(4) $$RMSE=\sqrt{\sum {\left(y_{i}-{\hat{y}}_{i}\right)}^{2}/n}$$

(5) $$MAE=\sum \left|y_{i}-{\hat{y}}_{i}\right|/n$$

(6) $$MAPE=(100\%/n)\times \sum \left|y_{i}-{\hat{y}}_{i}\right|/\left|y_{i}\right|$$

where $y_{i}$ is the measured value, ${\hat{y}}_{i}$ is the predicted value, ȳ is the mean of the measured values, and *n* is the sample size. *R*^2^ quantifies the proportion of variance explained by the model; *RMSE* and *MAE* measure the absolute magnitude of prediction error; and *MAPE* measures relative prediction error.

### 2.4. Interpretability Analysis Using SHAP and ALE

SHAP (SHapley Additive exPlanations) is a model-interpretability method based on cooperative game theory [36]. It assigns a Shapley value to each feature to represent that feature’s marginal contribution to a model prediction. For feature *j* in sample *i*, the Shapley value is defined as:

(7) $$\varphi_{j}(i)=\sum_{\left\{S\subseteq N\backslash \left\{j\right\}\right\}}\left|S\right|!(\left|N\right|-\left|S\right|-1)!/\left|N\right|!\times \left[f(S\cup \left\{j\right\})-f(S)\right]$$

where *N* is the complete feature set, *S* is a subset that does not contain feature *j*, and *f*(*S*) is the model prediction based on feature subset *S*. SHAP values have three important properties: (1) local accuracy—the sum of the Shapley values equals the difference between the model prediction and the baseline value; (2) consistency—the Shapley value of a feature does not decrease when its contribution across all samples increases; and (3) additivity—the Shapley values of multiple models can be combined linearly.

In addition to SHAP, accumulated local effects (ALE) were used to independently verify feature effects. ALE is a binning-based interpretation method that estimates the independent effect of a feature on model output by calculating the average difference in predictions at adjacent interval boundaries and accumulating these differences across intervals [37]. Unlike partial dependence plots (PDPs), ALE reduces interference from correlations among features and therefore more accurately represents the direction and magnitude of a feature’s main effect. It is particularly useful for identifying nonmonotonic relationships and assessing the robustness of feature-dependence patterns. Centered ALE values use zero as the baseline: positive values indicate that a feature value contributes positively to the model output, negative values indicate a negative contribution, and the absolute value indicates effect magnitude. All 300 samples were used to calculate ALE values separately for each of the 13 input features. The number of bins was set to 20, and quantiles were used to define the interval boundaries so that the samples were distributed evenly among bins.

### 2.5. Optimization Algorithm

The grey wolf optimizer (GWO) mimics the social hierarchy and hunting behavior of grey wolf packs. It offers several advantages, including parameter-free and derivative-free optimization, conceptual simplicity, ease of implementation, adaptability, flexibility, and robustness [38]. The GWO algorithm was implemented using the mealpy library (version 3.0.0) in Python 3.9, which provides a robust and efficient implementation of the metaheuristic optimization algorithm. The population is divided into four hierarchical levels: α (the best solution or global best individual), β (the second-best solution), δ (the third-best solution), and ω (all remaining individuals). The optimization process consists of three phases:

(1) Search phase: The grey wolves explore the solution space through random walks. In the algorithm, this corresponds to global search when A > 1.

(2) Encirclement phase: The grey wolves approach the prey, and their positions are updated as follows:

(8) $$D=\left|CX_{p}(t)-X(t)\right|$$

(9) $$X(t+1)=X_{p}(t)-A\cdot D$$

where $X_{p}$ represents prey position, $A=2a\cdot r_{1}-a$, $C=2r_{2}$, a decreases linearly from 2 to 0, and $r_{1}$ and $r_{2}$ are random numbers in [0, 1].

(3) Attack phase: When *A* < 1, the grey wolves approach and attack the three leading solutions, *α*, *β*, and *δ*:


(10) $$X(t+1)=\left[X_{\alpha }-A_{1}\left|C_{1}X_{\alpha }-X\right|+X_{\beta }-A_{2}\left|C_{2}X_{\beta }-X\right|+X_{\delta }-A_{3}\left|C_{3}X_{\delta }-X\right|\right]/3$$

The trained CatBoost forward-prediction model was used as a surrogate, and the weighted deviation between the predicted and target values was defined as the fitness function. GWO then searched for the optimal solution within the feasible domain of the mixture design. The population size N was set to 30, and the maximum number of iterations T was set to 300. Equal weights of 0.5:0.5 were assigned to MSR and TSR so that both indicators were considered equally. For specific engineering applications, these weights can be adjusted to reflect project requirements.

### 2.6. IMD Method

**Figure 1:**
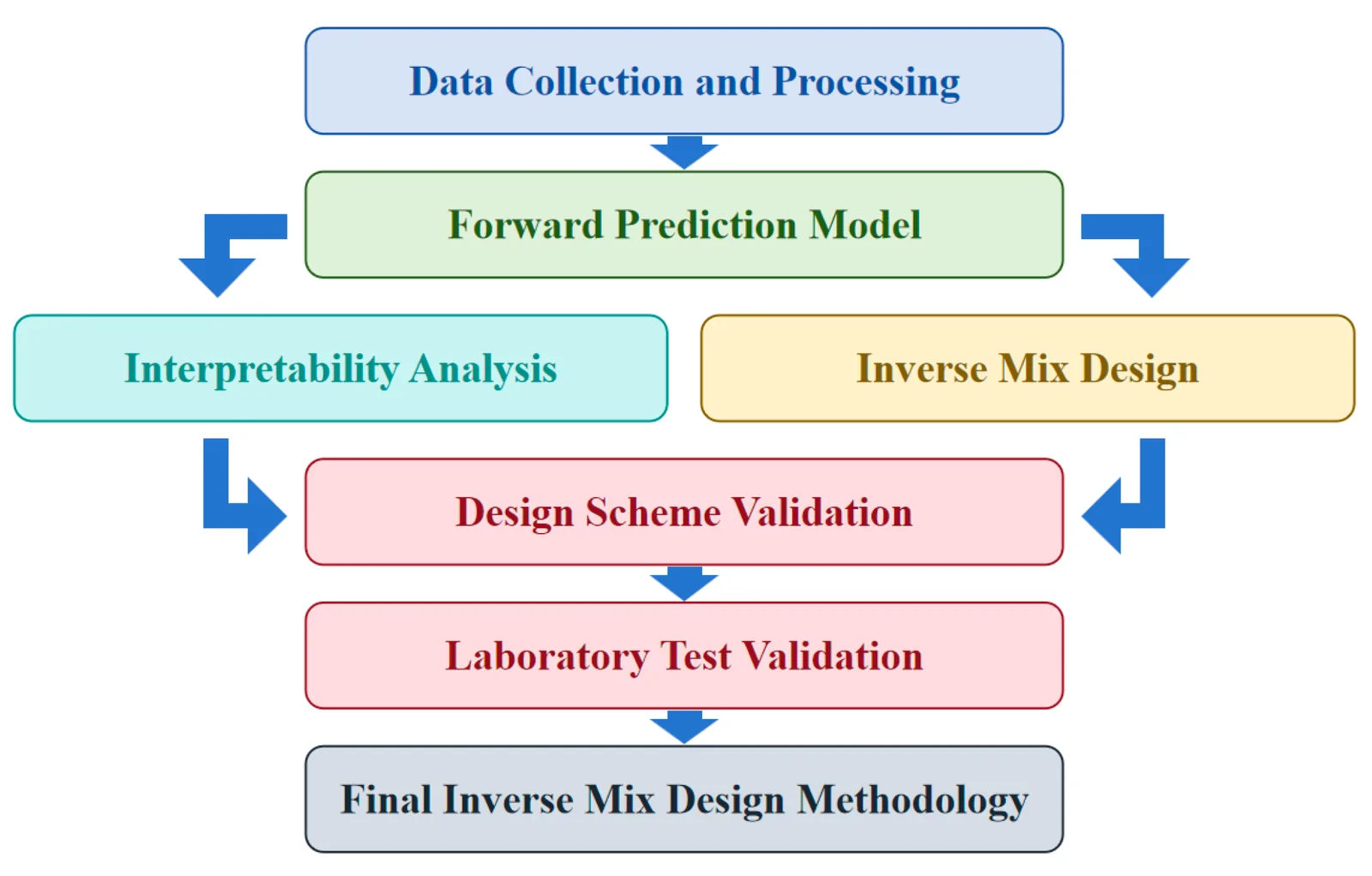
Flowchart of the IMD method.

The basic principle of inverse design is to determine the optimal combination of mix design parameters for specified performance targets. It can be formulated mathematically as follows:

(11) $$minF(x)=0.5\times \left|{MS}_{pred}(x)-{MS}_{target}\right|/{MS}_{target}\;\;+0.5\times \left|{TSR}_{pred}(x)-{TSR}_{target}\right|/{TSR}_{target}$$

where *x* represents the mix design variable vector (6-dimensional), including asphalt–aggregate ratio, air voids, passing rate at 13.2 mm, 9.5 mm, and 4.75 mm, mineral filler content, and steel slag replacement ratio; ${MS}_{pred}(x)$ and ${TSR}_{pred}(x)$ represent *MSR* and *TSR* predicted by the CatBoost model. Raw material properties such as asphalt penetration, softening point, asphaltene content, aggregate basicity, and angularity index, along with f-CaO content, were input as fixed scenario parameters and excluded from optimization search. Constraint conditions were determined based on specifications and engineering experience: asphalt–aggregate ratio 4.8~6.8%, air voids 3.0~5.0%, passing rate at 13.2 mm 90~100%, passing rate at 9.5 mm 55~78%, passing rate at 4.75 mm 18~42%, steel slag replacement ratio 0~100%. The IMD methodology flow is illustrated in Figure 1.

Raw material properties such as asphalt penetration, softening point, asphaltene content, aggregate basicity, angularity index, and f-CaO content were treated as fixed scenario parameters and excluded from the optimization search space. This design decision was based on the following rationale: (1) in engineering practice, raw material properties are determined by the material source and cannot be modified during proportion design; the mix design variables are optimized given these fixed material conditions, and the proposed framework mirrors this practical workflow; (2) treating these properties as scenario parameters enables the framework to be readily adapted to different material sources and project conditions; (3) some raw material properties are intrinsic characteristics that cannot be freely adjusted within arbitrary ranges, and including them as optimization variables could lead to physically unrealistic solutions. A potential future extension would be a two-level optimization framework with an outer loop to select optimal material combinations and an inner loop to optimize mixture proportions.

## 3. Results and Discussion

### 3.1. Comparison of Forward Prediction Model Performance

The test-set performance of the three models in predicting MSR and TSR is presented in Table 2.

Table 2 summarizes the test-set predictions of the three models. All models achieved R^2^ values above 0.80, indicating that they adequately captured the nonlinear relationships between material parameters and moisture susceptibility. RF performed best for MSR, although the differences among the three models were relatively small, whereas CatBoost achieved the best performance for TSR.

**Figure 2:**
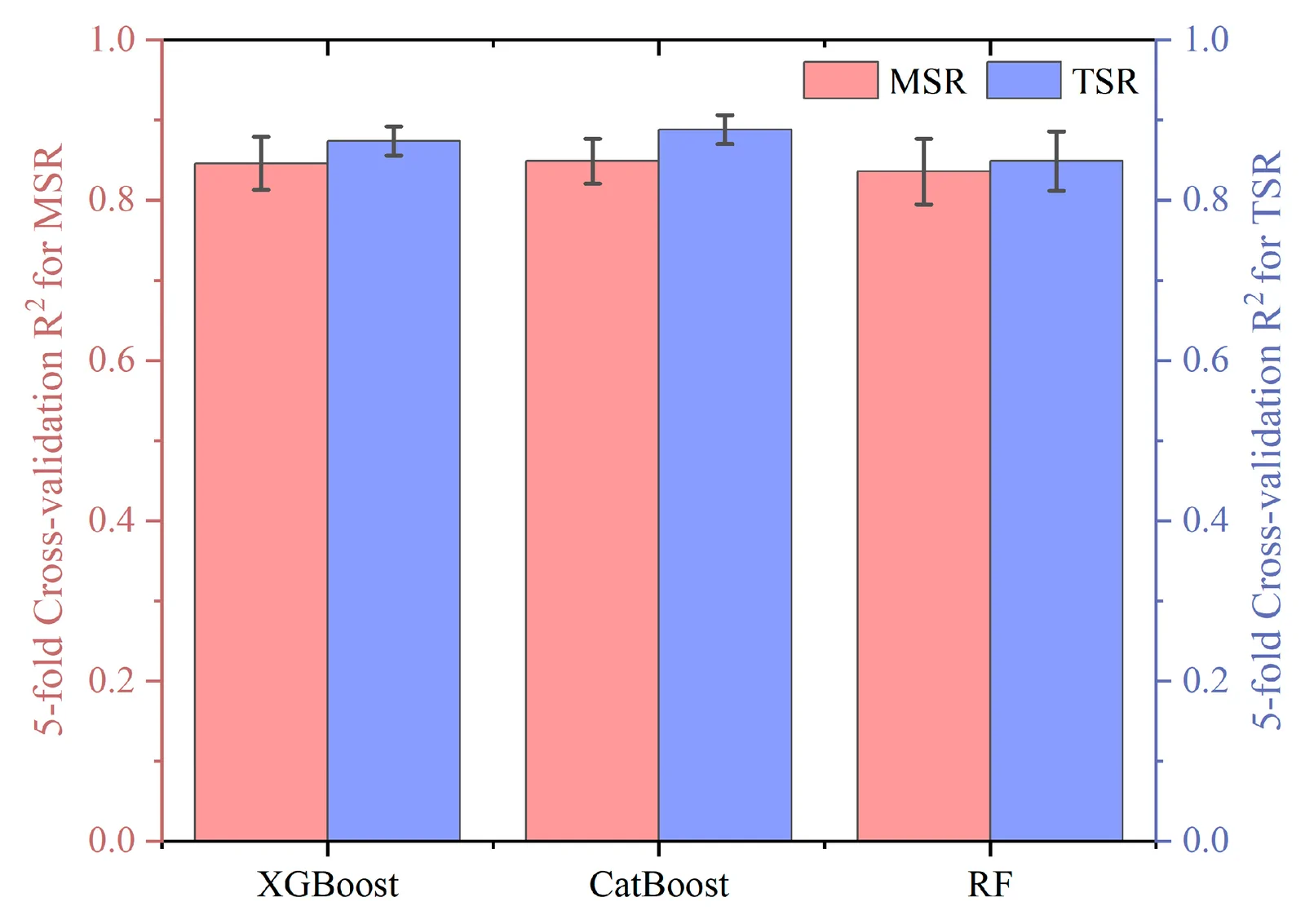
Fivefold cross-validation results.

The cross-validation results are presented in Figure 2. The fivefold R^2^ values obtained by CatBoost for MSR and TSR were 0.849 ± 0.028 and 0.888 ± 0.018, respectively, indicating high accuracy and low variability. XGBoost ranked second, followed by RF, and all three models showed satisfactory stability. Considering test-set performance, cross-validation robustness, and computational efficiency, CatBoost was selected as the surrogate model.

Although RF slightly outperformed CatBoost for two MSR metrics (MSR-RMSE: 1.899 vs. 1.932; MSR-MAE: 1.580 vs. 1.639), CatBoost was selected as the surrogate model for the following reasons: (1) CatBoost achieved substantially better TSR prediction across all four metrics (R^2^ = 0.918 vs. 0.891; RMSE = 1.437 vs. 1.661; MAE = 1.179 vs. 1.417), which is particularly important given the complexity of freeze–thaw mechanisms; (2) CatBoost achieved the highest 5-fold CV mean R^2^ for both MSR (0.849 ± 0.028) and TSR (0.888 ± 0.018), indicating the most stable generalization; and (3) CatBoost’s ordered boosting and symmetric tree structure reduce gradient estimation bias and improve numerical stability for engineering problems with mixed data types.

### 3.2. SHAP Global Feature Importance Analysis

**Figure 3:**
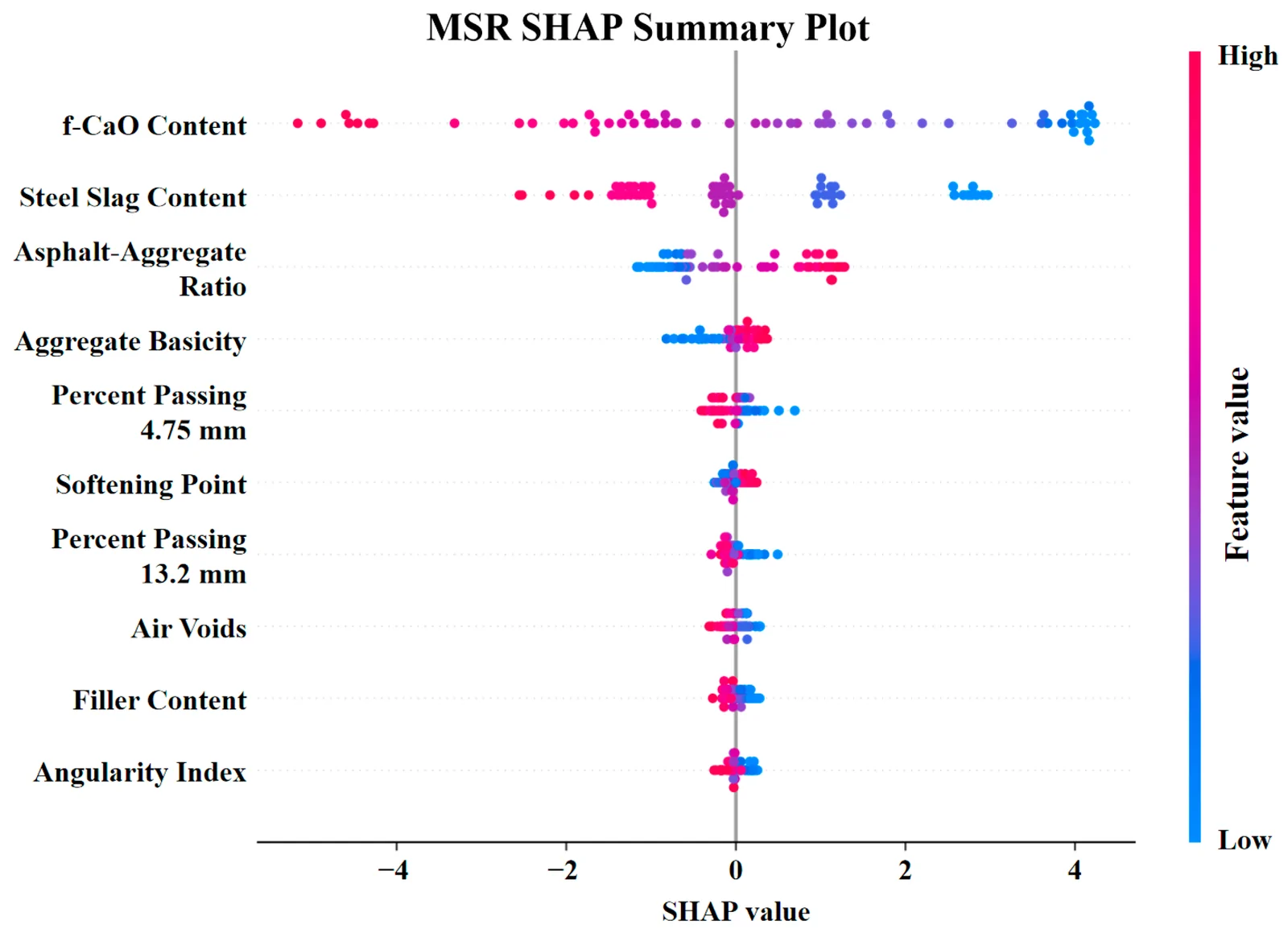
SHAP summary plot for MSR.

Figure 3 presents the SHAP summary plot for MSR, and Table 3 lists the five most important features. The f-CaO content and steel slag replacement ratio ranked first and second, respectively, and together accounted for more than 65% of the total importance. When exposed to water, f-CaO hydrates to form Ca(OH)_2_, causing volumetric expansion that directly compromises the volumetric stability of the steel slag and, under severe conditions, may damage the internal mixture structure and accelerate moisture damage. A higher steel slag replacement ratio increases the total amount of f-CaO in the mixture, thereby reducing moisture resistance. The asphalt–aggregate ratio ranked third because a higher asphalt content promotes the formation of thicker asphalt films and strengthens asphalt–aggregate adhesion. Aggregate basicity ranked fourth, reflecting the role of aggregate acid–base properties in adhesion: alkaline aggregates react with acidic asphalt components to form strong chemical bonds.

**Figure 4:**
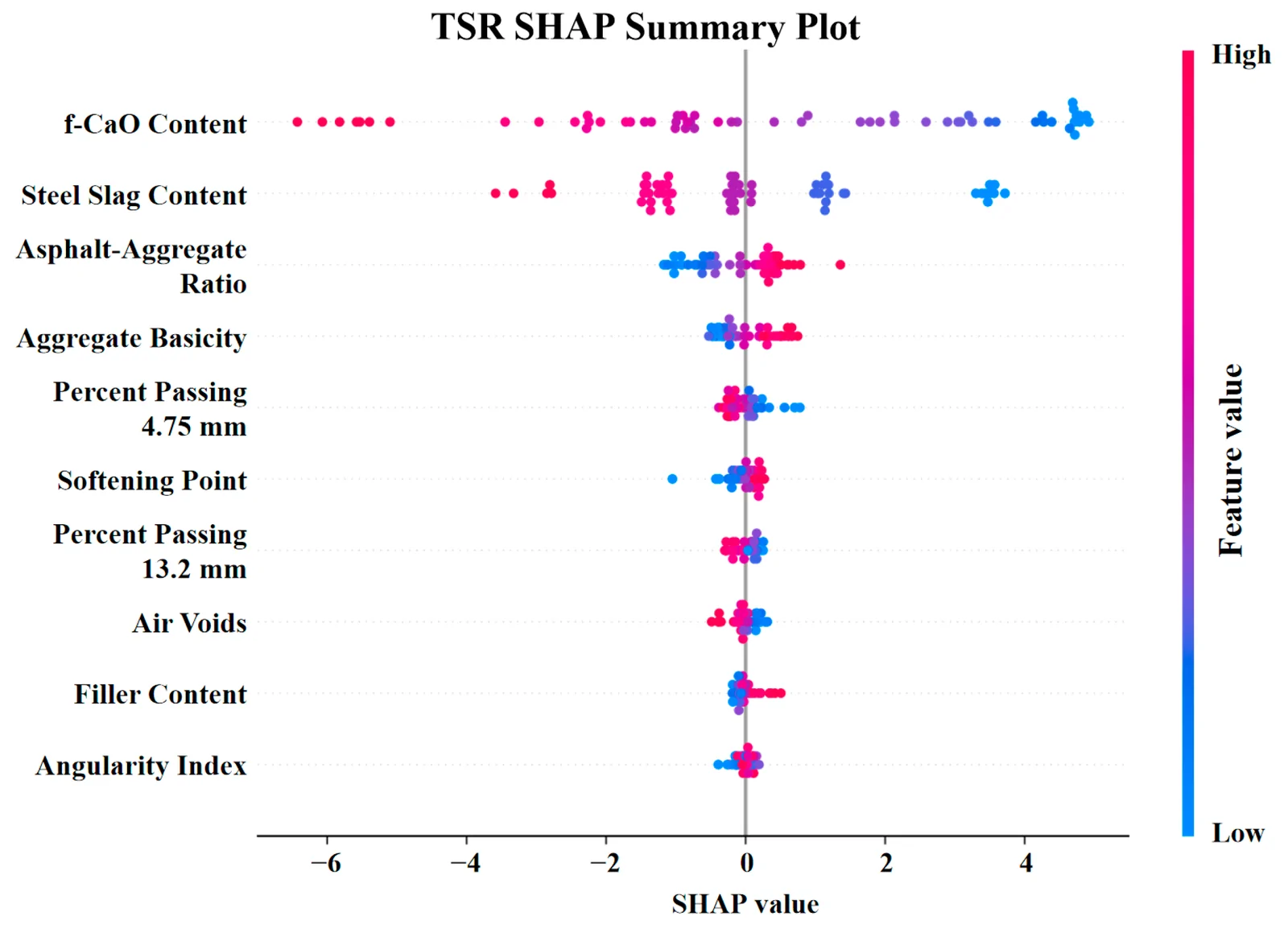
SHAP summary plot for TSR.

Figure 4 presents the SHAP summary plot for TSR, and Table 4 lists the five most important features. The importance of f-CaO content was even greater for TSR, indicating that TSR is more sensitive to variations in f-CaO. This finding is consistent with the physical mechanism by which freeze–thaw cycles amplify the effects of f-CaO hydration-induced expansion. Notably, asphaltene content ranked third for TSR. Freeze–thaw cycles involve repeated temperature fluctuations, and asphalt with a higher asphaltene content has greater viscosity and adhesion, allowing it to retain more bonding strength under freeze–thaw conditions.

### 3.3. Accumulated Local Effects Analysis

**Figure 5:**
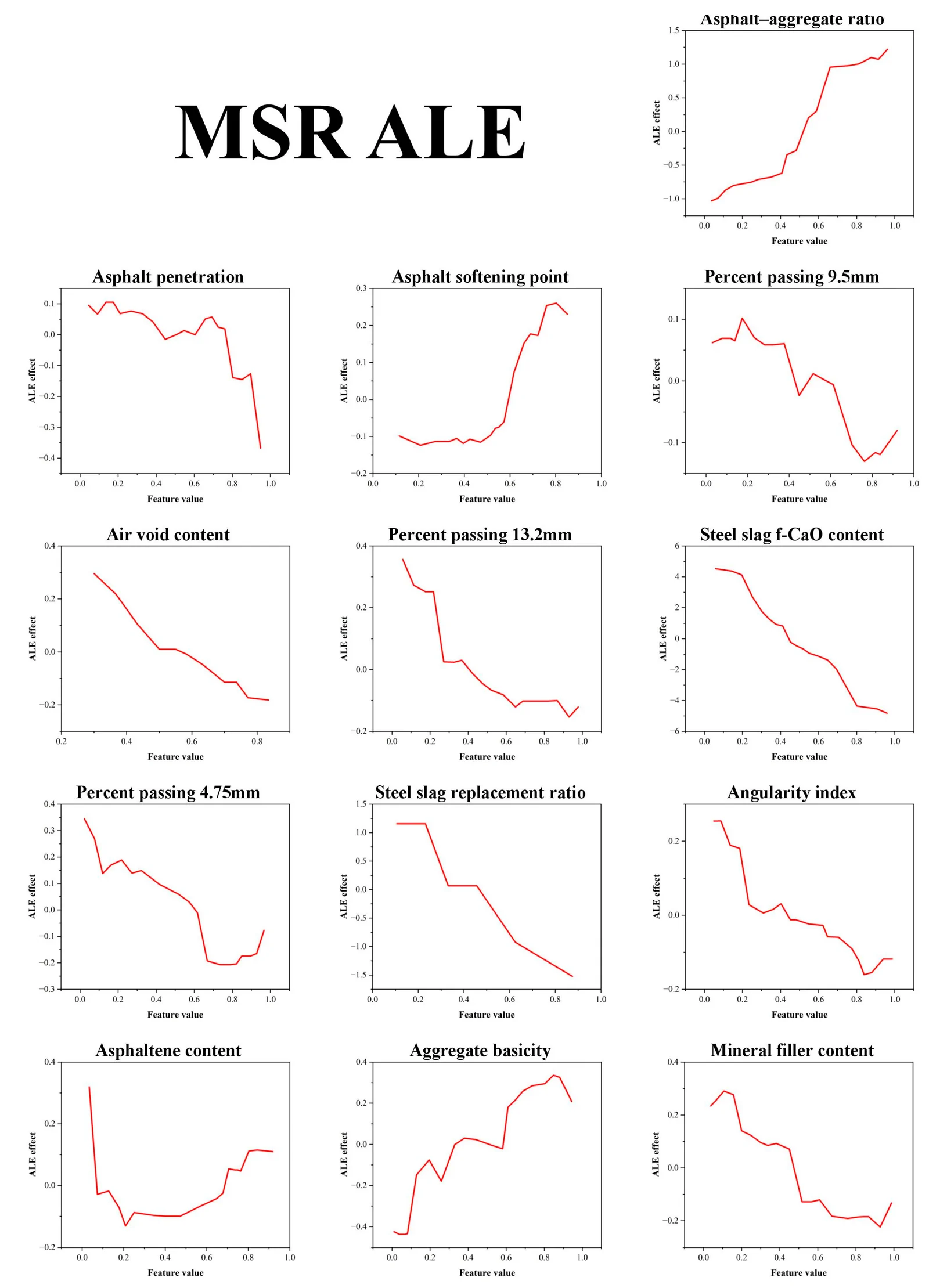
ALE plot for MSR.

**Figure 6:**
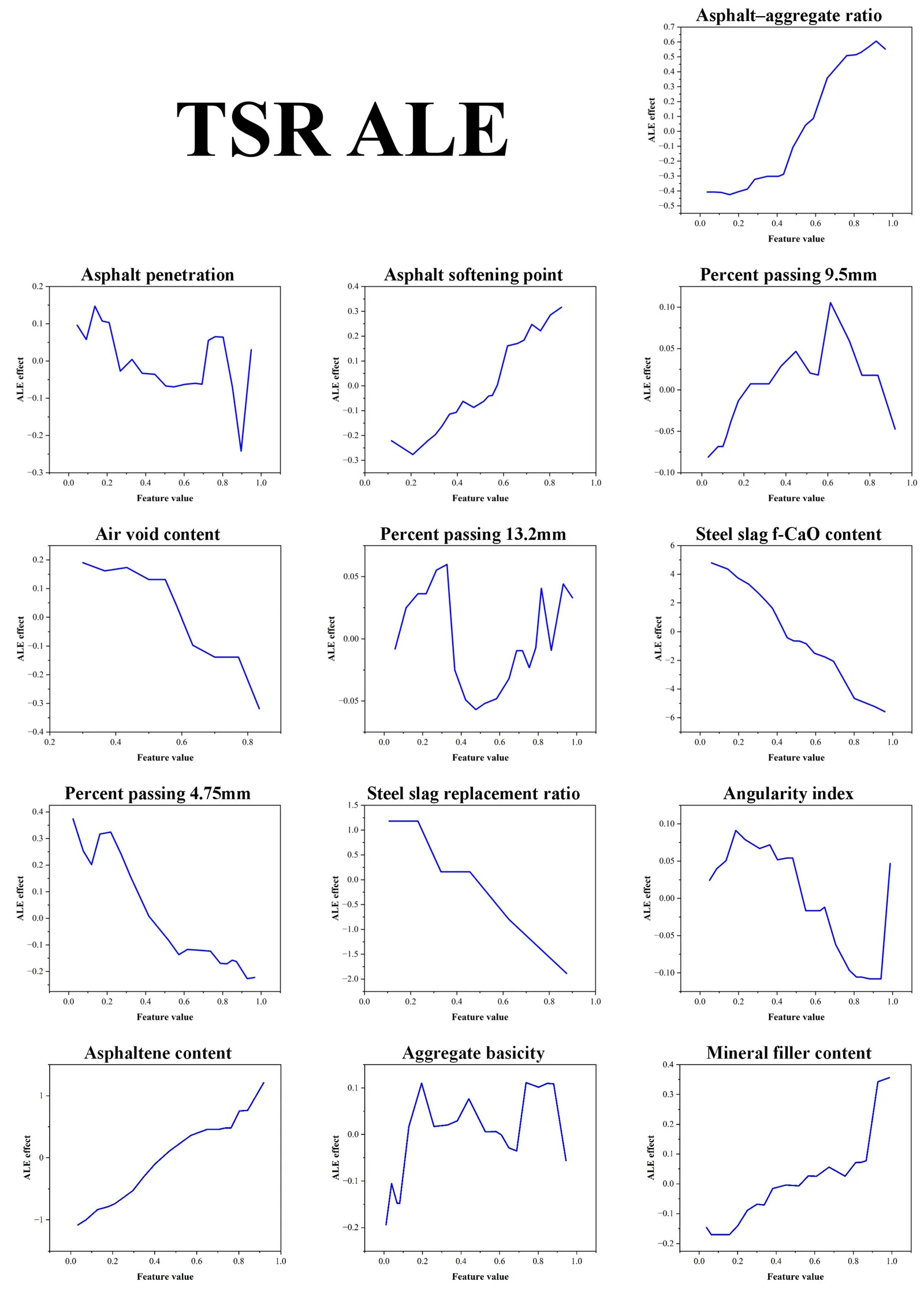
ALE plot for TSR.

SHAP analysis provided feature-importance rankings and dependence trends, but these results required independent verification. ALE was therefore used to analyze each of the 13 input features. Figure 5 and Figure 6 present the ALE results for the MSR and TSR models, respectively.

Figure 5 and Figure 6 show the ALE curves for MSR and TSR, respectively. Overall, the ALE trends for individual features were consistent with the preceding SHAP dependence analysis. Among the features, f-CaO content had a pronounced monotonic negative effect on both MSR and TSR. Its ALE value declined from approximately +4 at low f-CaO contents to approximately −6 at high contents, a range far greater than that of any other feature, confirming f-CaO content as the primary factor controlling moisture susceptibility. Once f-CaO content exceeded approximately 2.5%, the ALE value changed from positive to negative and declined more rapidly, indicating that the adverse effect of f-CaO hydration-induced expansion intensified beyond this threshold. The ALE curve for the steel slag replacement ratio also decreased monotonically, with a steeper decline above 75%, consistent with the 75% inflection point identified by the preceding SHAP analysis. For the asphalt–aggregate ratio, the ALE values for both MSR and TSR increased monotonically from approximately −1.5 to approximately +1.5. This trend indicates that a moderate increase in the asphalt–aggregate ratio improves moisture resistance by producing thicker asphalt films and stronger aggregate adhesion. The ALE curve for aggregate basicity was nonmonotonic and peaked at approximately 4.0–4.5; the lower ALE values at both low and high basicity indicate that excessive or insufficient basicity is unfavorable. Asphaltene content also had a marked positive effect on TSR, with ALE values increasing from approximately −0.8 to approximately +1.2, indicating that asphalt with a higher asphaltene content better retains bonding strength under freeze–thaw conditions. The ALEs of the three sieve passing rates were relatively small and followed different trends. This suggests that gradation has a limited independent effect on moisture susceptibility and that the passing rate of each sieve can be adjusted relatively independently within the specification limits. Overall, the ALE results confirmed the SHAP findings: f-CaO content and steel slag replacement ratio were the main factors affecting moisture susceptibility, followed by the asphalt–aggregate ratio and aggregate basicity, whereas gradation-related features had relatively small independent effects. Because ALE captures only the independent main effect of each feature and not interactions among features, a correlation matrix of SHAP values was subsequently used to characterize potential coupling among feature effects.

### 3.4. SHAP Value Correlation Analysis

**Figure 7:**
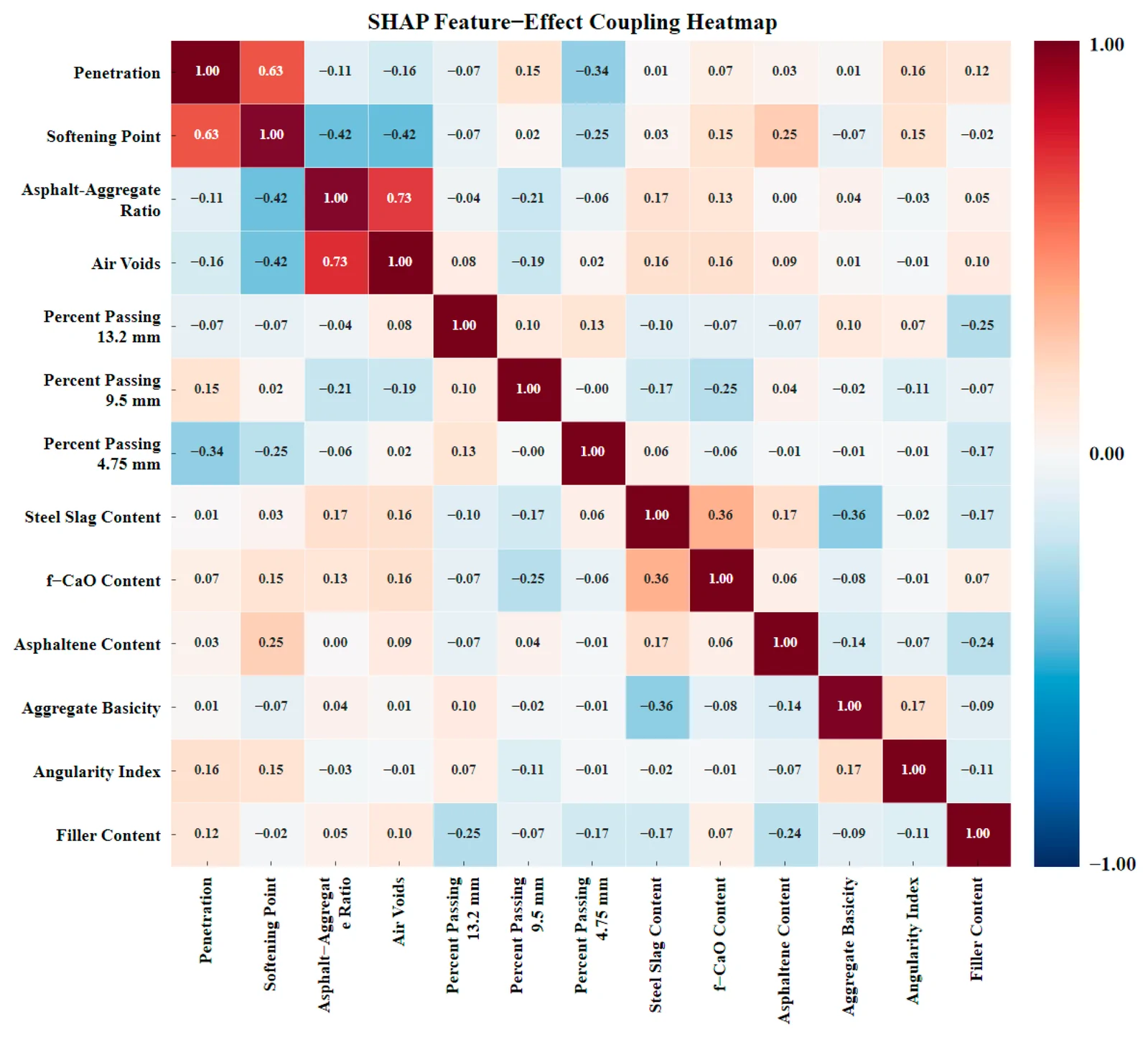
Heatmap of correlations among SHAP values.

The preceding ALE analysis confirmed the independent main effects of individual features; this section examines potential interactions among them. Correlation coefficients were calculated among the SHAP values and plotted as a heatmap. Global feature importance quantifies the independent contribution of each feature, whereas correlation analysis can identify features whose effects may act synergistically or antagonistically, thereby providing additional insight into multifactor coupling. Correlations among SHAP values indicate only whether two features affect the prediction in similar directions; they do not, by themselves, establish a rigorous interaction between the features. As shown in Figure 7, the correlation coefficient between the SHAP values of f-CaO content and steel slag replacement ratio exceeded 0.4, indicating a clear positive correlation. Thus, the adverse effect of f-CaO expansion became more pronounced as the steel slag replacement ratio increased. The asphalt–aggregate ratio and air voids were negatively correlated: increasing the asphalt–aggregate ratio reduced the air-void content, and their combined effect on moisture susceptibility was smaller than the sum of their individual effects. Accordingly, practical design should consider changes in air voids together with adjustments to the asphalt–aggregate ratio. Finally, the SHAP values of asphalt penetration and softening point were strongly correlated, indirectly supporting the use of empirical relationships to estimate the softening point from penetration.

### 3.5. Inverse Design Results

Using the trained CatBoost forward-prediction model as a surrogate, GWO was applied to inversely optimize the mixture proportions. Three validation scenarios with different performance targets were defined to represent different applications: Scenario 1 represented high-demand conditions, specifically the upper surface course of major arterial highways; Scenario 2 represented moderate-demand conditions on ordinary arterial highways; and Scenario 3 represented low-demand conditions on secondary roads or under specific engineering conditions. All target values met the current specifications for the moisture susceptibility of asphalt mixtures. 

With the surrogate model and the defined performance targets, GWO was employed to optimize six mix design variables: asphalt–aggregate ratio, air voids, 13.2 mm passing rate, 9.5 mm passing rate, 4.75 mm passing rate, and steel slag replacement ratio. The fitness function was defined as the weighted deviation between the CatBoost-predicted and target values for MSR and TSR. Raw material features, including f-CaO content, were treated as fixed scenario parameters.

The optimized mixture proportions are presented in Table 5.

Table 5 presents the inverse-designed mixture proportions for the three scenarios at different f-CaO levels. Raw material features, including f-CaO content, were treated as fixed scenario parameters, and six mix design variables were optimized. Under the low-f-CaO condition, the optimal steel slag replacement ratio was 49.48%, allowing substantial use of steel slag. Under the moderate-f-CaO condition, it was limited to 8.84%, demonstrating the strong constraint imposed by f-CaO content. Under the relatively high-f-CaO condition, the optimal replacement ratio was 13.70%, which was higher than that of Scenario 2. This was because Scenario 3 had relatively relaxed performance targets, imposing less stringent requirements on moisture susceptibility and therefore permitting greater steel slag utilization. These results are consistent with the SHAP analysis: f-CaO content was the most important factor affecting moisture susceptibility. At low f-CaO contents, the adverse effect of the steel slag replacement ratio on moisture resistance was substantially reduced, allowing higher replacement ratios.

To verify whether the targets could be achieved by these designs, the trained CatBoost model was used to predict the performance of each mixture in Table 5. The predicted and target values are compared in Table 6.

**Figure 8:**
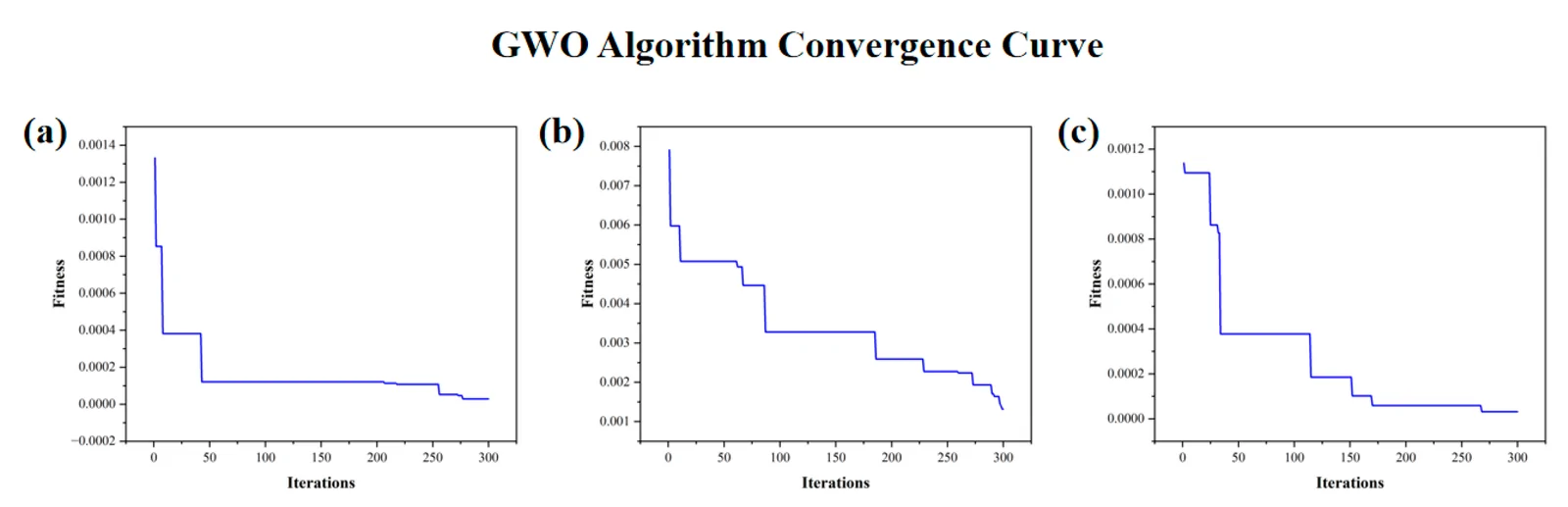
Convergence curves of the GWO algorithm: (**a**) Scenario 1; (**b**) Scenario 2;(**c**) Scenario 3.

As shown in Figure 8, the GWO fitness value approached the optimum within 30–50 iterations; subsequent iterations further refined the solutions, which converged fully within 300 iterations. Table 6 summarizes the GWO results for the three scenarios. The search error was below 0.24% in every scenario, and convergence was rapid. The predicted values for Scenarios 1 and 3 were nearly identical to their targets. In Scenario 2, the errors were 0.21% for MSR and 0.02% for TSR. The targets were achievable in all three scenarios, confirming the effectiveness of the optimization.

### 3.6. Laboratory Validation

**Figure 9:**
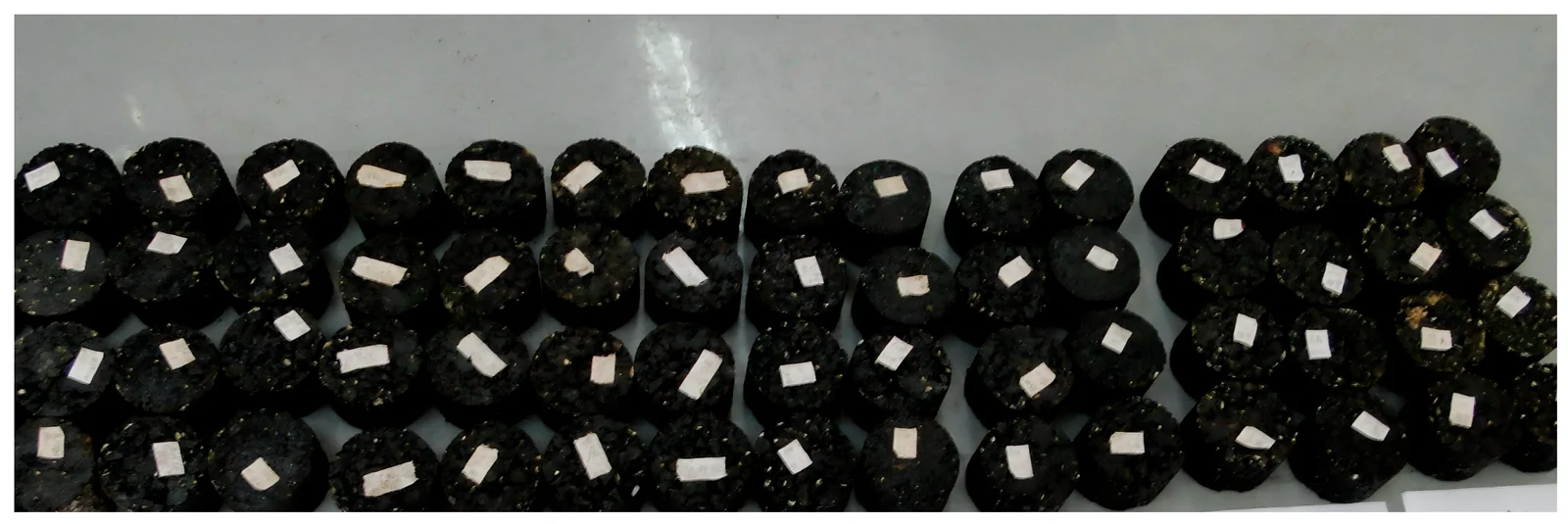
Marshall specimens used for laboratory validation.

To evaluate the engineering applicability of the proposed method, laboratory validation tests were conducted using the mixture proportions listed in Table 7. The validation tests used the same batches of raw materials as the training dataset. Twenty specimens were prepared for each mixture design and tested separately for MSR and TSR. After outliers were excluded, the arithmetic mean of the valid results was taken as the measured value. The f-CaO content of the steel slag was verified by chemical titration and was consistent with the target value. The Marshall specimens are shown in Figure 9, and the validation results are presented in Table 7.

The validation results in Table 7 show that the deviations between the target and measured values were no greater than 2.11% across the three scenarios. For Scenario 1, the deviations in MSR and TSR were 0.75% and 0.65%, respectively, reflecting the higher model accuracy and more comprehensive data coverage under low-f-CaO conditions. Scenario 2 produced slightly larger deviations, although the performance remained satisfactory. Scenario 3 had the smallest deviations, demonstrating the reliability of the method under relatively high-f-CaO conditions and conservative performance targets. Across all three scenarios, the mean of the six deviations was 1.02%, and every deviation satisfied the engineering accuracy requirement of less than 5%. These results confirm the physical soundness and engineering feasibility of the inverse designs.

## 4. Conclusions

This study developed a machine-learning-based IMD method for steel slag asphalt mixtures. The main conclusions are as follows:

(1) A comprehensive dataset for steel slag asphalt mixtures was compiled, comprising 300 samples, 13 input features, and 2 output indicators. A comparison of XGBoost, CatBoost, and RF showed that all three ensemble-learning models performed well. CatBoost provided the best overall prediction performance, with 5-fold cross-validation R^2^ values of 0.849 ± 0.028 for MSR and 0.888 ± 0.018 for TSR, along with stable generalization performance, and was therefore selected as the surrogate model for IMD.
(2) SHAP analysis identified f-CaO content and steel slag replacement ratio as the two dominant factors for both MSR and TSR. For MSR, the asphalt–aggregate ratio ranked third, followed by aggregate basicity. For TSR, asphaltene content ranked third, reflecting freeze–thaw sensitivity, and f-CaO exhibited an even greater influence due to amplification by freeze–thaw cycles. SHAP value correlation analysis confirmed consistent influence directions between f-CaO content and replacement ratio, and a negative correlation between asphalt–aggregate ratio and air voids. ALE analysis independently verified these feature effects.
(3) The GWO search error was below 0.24% in all three inverse design scenarios. Near-optimal solutions were obtained within approximately 30–50 iterations, and full convergence was achieved within 300 iterations. When f-CaO content was treated as a fixed scenario parameter, the optimal mixture proportions varied substantially among f-CaO levels: a high steel slag replacement ratio was feasible at low f-CaO contents, whereas a lower replacement ratio was required at high f-CaO contents. Laboratory validation yielded a mean deviation of 1.02% between the measured and target values, confirming the accuracy and reliability of the method.
(4) The current framework treats raw material properties as fixed scenario parameters, aligning with engineering practice but limiting the optimization scope. Future work will extend the framework that simultaneously considers material selection and proportion design, and incorporate additional performance indicators (e.g., Marshall stability, fatigue resistance, permanent deformation) for multi-objective optimization as corresponding experimental data become available. Moreover, by enabling performance-driven optimization of steel slag asphalt mixtures, the proposed method facilitates higher utilization rates of steel slag while ensuring pavement performance, thus contributing to CO_2_ emission reduction, natural aggregate conservation, and the circular economy in pavement infrastructure.

## Figures and Tables

**Table 1:** Description of the input features in the dataset.

No.	Feature	Unit	Range	Mean	Std. Dev.	Description
1	Asphalt penetration (25 °C)	0.1 mm	35–80	57.3	13.3	Characterizes asphalt consistency and temperature susceptibility
2	Asphalt softening point	°C	42–55	46.4	2.2	Characterizes high-temperature performance
3	Asphalt–aggregate ratio	%	4.5–7.0	5.8	0.6	Core mix proportion parameter
4	Air voids	%	2.5–7.0	4.2	0.3	Key volumetric index
5	13.2 mm passing rate	%	90–100	95.5	2.0	Controls the nominal maximum size
6	9.5 mm passing rate	%	45–78	65.0	5.8	Primary sieve controlling the aggregate skeleton
7	4.75 mm passing rate	%	18–42	29.0	5.2	Boundary between fine and coarse aggregate
8	Steel slag replacement ratio	%	0–100	51.2	32.4	Volumetric replacement ratio of coarse aggregate
9	Steel slag f-CaO content	%	0–5.5	2.1	1.4	Key indicator of volumetric stability
10	Asphaltene content	%	11–20	15.5	2.6	Affects asphalt adhesion
11	Aggregate basicity	-	2.5–5.5	4.0	0.9	Acid–base index of aggregate
12	Angularity index	-	0.75–1.15	0.94	0.12	Aggregate morphology
13	Mineral filler content	%	6–10	8.0	1.2	Filling effect

**Table 2:** Comparison of predictive performance among the three models.

Model	MSR-R^2^	MSR-RMSE (%)	MSR-MAE (%)	MSR-MAPE (%)	TSR-R^2^	TSR-RMSE (%)	TSR-MAE (%)	TSR-MAPE (%)
XGBoost	0.837	1.853	1.561	1.829	0.887	1.695	1.414	1.796
CatBoost	0.823	1.932	1.639	1.921	0.918	1.437	1.179	1.499
RF	0.829	1.899	1.580	1.846	0.891	1.661	1.417	1.805

**Table 3:** SHAP feature-importance ranking for MSR (top 5).

Rank	Feature	SHAP Importance	Proportion
1	f-CaO content	2.4435	44.1%
2	Steel slag replacement ratio	1.2147	21.9%
3	Asphalt–aggregate ratio	0.7468	13.5%
4	Aggregate basicity	0.2179	3.9%
5	4.75 mm passing rate	0.1646	3.0%

**Table 4:** SHAP feature-importance ranking for TSR (top 5).

Rank	Feature	SHAP Importance	Proportion
1	f-CaO content	2.8984	47.3%
2	Steel slag replacement ratio	1.4335	23.4%
3	Asphaltene content	0.5168	8.4%
4	Asphalt–aggregate ratio	0.338	5.5%
5	4.75 mm passing rate	0.1889	3.1%

**Table 5:** Mixture proportions obtained by inverse design.

Scenario	f-CaO (%)	Asphalt-Aggregate Ratio	Air Voids	13.2 mm Passing	9.5 mm Passing	4.75 mm Passing	Steel Slag Ratio	Penetration	Softening Point	Asphaltene Content
1	0.51	6.37	3.24	97.10	74.98	18.52	49.48	63.7	45.3	19.70
2	2.16	5.59	3.67	98.71	59.28	32.64	8.84	41.3	49.8	13.73
3	3.19	4.91	3.77	94.33	75.12	20.69	13.70	36.1	49.6	12.42

**Table 6:** GWO results.

Scenario	Target MSR (%)	Target TSR (%)	Predicted MSR (%)	Predicted TSR (%)
1	90.00	85.00	90.13	85.06
2	87.00	83.00	87.21	82.98
3	83.00	77.00	82.98	79.95

**Table 7:** Laboratory validation results.

Scenario	Indicator	Target Value	Measured Mean	Deviation Between Target and Measured Values
1	MSR (%)	90.00	89.33 ± 1.39	0.75%
	TSR (%)	85.00	84.45 ± 1.57	0.65%
2	MSR (%)	87.00	88.56 ± 1.29	1.52%
	TSR (%)	83.00	84.77 ± 1.53	2.11%
3	MSR (%)	83.00	82.65 ± 0.60	0.42%
	TSR (%)	77.00	76.65 ± 1.06	0.45%

## Data Availability

The original contributions presented in this study are included in the article. Further inquiries can be directed to the corresponding author.
